# Options for Active Case Detection of Visceral Leishmaniasis in Endemic Districts of India, Nepal and Bangladesh, Comparing Yield, Feasibility and Costs

**DOI:** 10.1371/journal.pntd.0000960

**Published:** 2011-02-08

**Authors:** Shri Prakash Singh, Siddhivinayak Hirve, M. Mamun Huda, Megha Raj Banjara, Narendra Kumar, Dinesh Mondal, Shyam Sundar, Pradeep Das, Chitra Kumar Gurung, Suman Rijal, C. P. Thakur, Beena Varghese, Axel Kroeger

**Affiliations:** 1 Department of Community Medicine, Institute of Medical Sciences, Banaras Hindu University, Varanasi, India; 2 Vadu Rural Health Program, KEM Hospital Research Center, Pune, India; 3 Parasitology, Laboratory Sciences Division, International Center for Diarrheal Diseases Research, Dhaka, Bangladesh; 4 Institute of Medicine, Tribhuvan University, Kathmandu, Nepal; 5 Rajendra Memorial Research Institute of Medical Sciences, Patna, India; 6 Department of Internal Medicine, BP Koirala Institute of Health Sciences, Dharan, Nepal; 7 Balaji Utthan Sanstha, Patna, India; 8 Public Health Foundation of India, New Delhi, India; 9 World Health Organization, Special Programme for Research and Training in Tropical Diseases, TDR-WHO, Geneva, Switzerland; 10 School of Tropical Medicine, Liverpool, United Kingdom; Institut Pasteur de Tunis, Tunisia

## Abstract

**Background:**

The VL elimination strategy requires cost-effective tools for case detection and management. This intervention study tests the yield, feasibility and cost of 4 different active case detection (ACD) strategies (camp, index case, incentive and blanket approach) in VL endemic districts of India, Nepal and Bangladesh.

**Methodology/Principal Findings:**

First, VL screening (fever more than 14 days, splenomegaly, rK39 test) was performed in camps. This was followed by house to house screening (blanket approach). An analysis of secondary VL cases in the neighborhood of index cases was simulated (index case approach). A second screening round was repeated 4–6 months later. In another sub-district in India and Nepal, health workers received incentives for detecting new VL cases over a 4 month period (incentive approach). This was followed by house screening for undetected cases. A total of 28 new VL cases were identified by blanket approach in the 1^st^ screening round, and used as ACD gold standard. Of these, the camp approach identified 22 (sensitivity 78.6%), index case approach identified 12 (sensitivity – 42.9%), and incentive approach identified 23 new VL cases out of 29 cases detected by the house screening (sensitivity – 79.3%). The effort required to detect a new VL case varied (blanket approach – 1092 households, incentive approach – 978 households; index case approach – 788 households had to be screened). The cost per new case detected varied (camp approach $21 – $661; index case approach $149 – $200; incentive based approach $50 – $543; blanket screening $112 – $629). The 2^nd^ screening round yielded 20 new VL cases. Sixty and nine new PKDL cases were detected in the first and second round respectively.

**Conclusions/Significance:**

ACD in the VL elimination campaign has a high yield of new cases at programme costs which vary according to the screening method chosen. Countries need the right mix of approaches according to the epidemiological profile, affordability and organizational feasibility.

## Author Summary

For the elimination of any infectious disease (i.e., reduction of the burden of a serious public health problem to a minor problem which can be managed by the general health services) the right mix of public health tools has to be identified for the early detection and successful treatment of new cases as well as effective vector control (in the case of vector borne diseases) at affordable costs. The paper provides a powerful example of evidence building for cost-effective early case detection in the visceral leishmaniasis elimination initiative of Bangladesh, India and Nepal. It compares the camp approach (mobile teams testing in chronic fever camps for spleen enlargement and rapid diagnostic tests) with the index case approach (screening for new cases in the neighbourhood of reported visceral leishmaniasis patients) and the incentive based approach (where basic health workers receive an allowance for detecting a new case) using subsequent house-to-house screening for the identification of the real number of un-detected cases. By applying a mix of different study methods and an itinerate research process to identify the most effective, feasible and affordable case detection method, under different environmental conditions, recommendations could be developed which help governments in shaping their visceral leishmaniasis elimination strategy.

## Introduction

India, Nepal and Bangladesh have committed to eliminate VL by 2015 and have adopted multiple public health strategies towards the elimination goal of reducing VL cases to less than 1 per 10,000 population [1]. Treatment with Miltefosine, the first oral effective drug, the possibility of an effective safe and affordable single dose liposomal amphotericin for treatment, the high specificity of rk39 rapid diagnostic test, and the absence of an animal host reservoir, makes VL a potential target disease for elimination [2]–[4].

VL control program strategies aim to reduce morbidity and mortality and involve a number of approaches. These include early diagnosis and prompt treatment of VL cases, surveillance for early detection of VL outbreaks, making available appropriate diagnostic facilities and drugs, promotion of health awareness, clinical and epidemiological research, and implementation of integrated vector management strategies including indoor residual spraying, use of bed-nets and improvement of environmental and housing conditions.

However, despite these efforts, VL transmission continues primarily in the absence of an effective surveillance system. Though VL patients ultimately report to health centers and hospitals, diagnosis is often missed in the early stages of infection and delayed due to lack of diagnostic facilities at peripheral levels of health systems with a consequent delay in treatment and sustaining the human reservoir [5], [6].

Shortage and supply delays of VL drugs, unavailability of diagnostics at the health center level often contributes to the community's poor public perception of the public health systems, a loss of faith and consequent low community participation/involvement in VL control program activities [7]. A passive surveillance for VL in the public sector is further hampered by an un-regulated private sector which fails to notify VL disease even where required by regulation.

This paper analyzes the feasibility, new case yield and costs of different active case detection strategies as applied in different country VL control program contexts at different VL endemicity levels.

## Materials and Methods

### Ethics Statement

The research program was approved by the Ethics Committees of all participating sites (Ethics Committee, Rajendra Memorial Research Institute of Medical Sciences; Ethics Committee, Institute of Medical Sciences, Banaras Hindu University; Institutional Review Board, Institute of Medicine, Tribhuvan University; Ethics Review Committee, ICDDR,B, and Ethics Review Committee, World Health Organization). Subjects participated in the study after a written informed consent.

### Study design

The study was designed to test 4 different ACD strategies – camp, index case and blanket ACD strategies (see below) implemented serially in one health center area; and incentives based ACD strategy implemented in parallel in a geographically discontinuous health center area. The study was conducted from May to December 2009.

### Study area profile

The study was conducted in the highly VL endemic districts of Saran and Muzaffarpur in Bihar in India, Sarlahi district of Nepal and Mymensingh district of Bangladesh. These regions were selected for their reported high annual VL incidence (per 10,000 population) varying from 20 – 25 in India, 5 – 8 in Nepal and 13 – 31 in Bangladesh. VL control strategies have been implemented variably in all the study districts. Indoor residual spraying has been sporadic and more focal in ‘hot spots’ both in the districts of India and Nepal but has not been done in Bangladesh. Miltefosine and amphotericin B was available in the study districts of India and Nepal but not yet in Bangladesh where at the time of the study, antimonials continued to be the mainstay of VL treatment. VL surveillance relied entirely on passive reporting.

### Active Case Detection strategies

The following 4 different ACD strategies were tested for early identification of VL cases in India, Nepal and Bangladesh:

### Camp approach (Mobile teams visiting target villages)

A camp schedule was developed by the researchers in consultation with District and Health Center Officials. Community sensitization meetings were conducted through village health workers at the village level to solicit involvement of village elders and leaders in the organization of the camps. The Health Center Medical Officer was invited to conduct/assist in the camp. Anganwadi workers, Accredited Social Health Activists (ASHAs in India), health workers and other governmental and non-governmental village level functionaries were assigned roles for promotion and conduct of the camps. Public announcements using loudspeakers were used one day before and on the day of the camp for ensuring publicity for the camps. During the camp, all attendees were screened for fever more than 14 days and examined for splenomegaly. Patients with a positive rK39 test were referred to the nearest health center for treatment with subsequent follow up at home. Additionally, patients with a history of VL treatment in the past and suspected PKDL-like skin lesions were tested with rK39 RDT and referred for confirmation of diagnosis and treatment. The duration of camps was between half to one day; in Nepal, due to the long travel distances, 3 camps in different villages were held back to back while in India and Bangladesh the camp staff returned to their base over night.

### Blanket approach (house to house screening)

House to house screening was used as a gold standard to assess how many new VL cases were missed by the camp approach. This was done including all households of the target villages the day following the camp. All households were numbered and members were screened by trained field staff for fever more than 14 days using a short screening questionnaire which also contained questions on VL disease which had occurred during the 12 months preceding the interview. Persons with chronic fever were examined for spleen enlargement by a physician/trained technician, and if positive were tested with rK39 and then sent for treatment to the closest health centre. Similarly, individuals with a past history of VL treatment with PKDL-like lesions were either referred to the health center or tested with rK39 test and if positive referred for confirmation of PKDL diagnosis and treatment.

### Index case based approach (search for new cases in the neighborhood of known cases)

To avoid a repeat survey in the same village following the blanket approach, the index case approach was simulated. A list of known VL patients in the target villages of the study treated in the past or currently on treatment was obtained from the health center records. These patients (defined in our study as ‘index’ case) were traced in the village and then visited in reality at their house where their presence was confirmed. All household identity numbers within 50 m radius or up to 100 households around the ‘index’ case were listed. This household list was then compared with the households screened in the blanket approach. Any individual from this household list, who had been detected by the house to house screening as a new VL case, was considered a new VL case detected by the index case approach.

### Incentives based approach (case detection by village health workers who received an incentive as additional motivation for detecting new VL cases)

This approach was implemented in India and Nepal (not in Bangladesh for political reasons) during a 4-month period in a well defined geographically distinct health center area. Health workers, Anganwadi workers, ASHA workers and community health volunteers were trained in the early identification and referral of suspected VL and PKDL cases in the community. Visual screening aids including photographs of PKDL cases were used for identifying patients for referral on an ongoing basis. An innovative incentive structure was developed in consultation with district health officials. Incentives (about USD 6) were paid for each new VL/PKDL case detected. All confirmed cases of VL/PKDL identified through this approach were ascertained by the researchers. After 4 months of incentive based ACD, a blanket house to house survey was conducted to ascertain the remaining VL/PKDL cases in the community not detected by the incentive based approach.

### Timeline and sequence of sub-studies

In order to assess the need for repeated camps in the same endemic villages, the camp approach with subsequent house to house screening was repeated 4 to 6 months after the first round in order to measure the yield of new cases after a certain period of time.

### Definitions and treatment strategies

The WHO definition of suspected VL (fever >14 days with splenomegaly in a VL endemic area and a positive rK39) was taken as basis for initiating treatment as indicated in the national treatment guidelines of the three countries. Patients with PKDL like skin lesions with a past history of VL treatment and testing positive for rK39 were treated for PKDL.

### Outcome measures and sample size

The different ACD strategies were evaluated for their sensitivity of detecting new VL cases using the blanket approach as the reference. Assuming a VL incidence of about 12 per 10,000 population, a total of about 48 newly diagnosed VL cases are expected to be detected per study site which requires a screening population of 40,000 individuals per site. This sample will detect an average sensitivity of 80% with 8% absolute precision at a.05 significance level. On the basis of this assessment, it was decided to screen a population of approximately 40,000 people per site.

### Cost analysis

In order to calculate the costs of different forms of active case detection for the control program, only those direct costs were included in our assessment which would have to be paid from the budget of the governmental VL control program. The following cost elements were assessed: training, local travel and costs for preparation, costs for materials and allowance costs for personnel involved in the approach.

### Data management and analysis

Data was entered in Epi Info at each site, preliminary cleaning was done before transferring the data base to the data management centre in Pune, India. A second check was performed and a joint data base was created. The data analysis was done using STATA.

## Results

### Background characteristics

The study was carried out in known VL endemic districts (annual incidence varying from 5 per 10,000 in Nepal to 30 per 10,000 in India and Bangladesh). The population in the study districts was poor and less educated compared to the national average. The age and sex composition of the study population across all sites was similar showing a relatively young population.

### ACD strategies for VL case detection

#### Camp approach

A total of 61 and 52 VL/PKDL screening camps were held in the 1^st^ and 2^nd^ round of ACD respectively. Overall, camps were more successful in screening for VL in Bangladesh and Nepal (average number of patients with fever for more than 14 days, detected at the camps 11.8 and 13.8 respectively) while camp screening outcome was lower at the Indian sites (average 9.7 chronic fever patients per camp). Out of a total of 345 chronic fever cases identified in the 1^st^ round, 22 had spleen enlargement and tested positive with rK39 test (3 in Muzarfurpur and 5 in Saran, India, 5 in Nepal and 9 in Bangladesh) (table 1). The percentage of chronic fever cases testing positive for VL was 6.4% with 3.4% and 11.1% in camps in India, 6.2% in Nepal and 6.8% in Bangladesh in the 1^st^ round of intervention.

**Table 1:** Case yield by different ACD strategies.

	Round 1					Round 2 (4 to 6 months after 1^st^ round)				
	SaranIndia	M'purIndia	SarlahiNepal	M'SinghB'desh	Overall	SaranIndia	M'purIndia	SarlahiNepal	M'SinghB'desh	Overall
*Camp approach*										
No. of Camps held	19	21	6	15	61	18	14	6	14	52
Average # of camp attendees	301	87	83	178	649	351	23	63	77	514
Old VL cases^1^ (<1 yr) detected	5	2	3	9	19	1	0	21	0	22
Fever cases (>14 days) screened	45	87	80	133	345	64	23	45	64	196
New VL cases^2^ detected (rK39 +ve)	5	3	5	9	22	5	2	3	2	12
Old PKDL cases (<1 yr) detected	1	0	0	1	2	0	0	0	0	0
Suspected Skin lesions screened	0	0	3	50	53	0	0	0	7	7
New PKDL cases detected	0	0	0	42	42	0	0	0	3	3
*Blanket approach*										
Total population screened	47908	42761	35081	40100	165850	48461	43565	33744	40065	165835
Old VL cases (<1 yr) detected	127	113	96	80	416	32	23	4	74	133
Fever cases (>14 days)	60	157	80	252	549	82	95	71	161	409
New VL cases^3^ detected (rK39 +ve)	6	3	5	14	28	8	2	3	7	20
Old PKDL cases^4^ (<1 yr) detected	8	3	0	13	24	0	4	1	17	22
Suspected Skin lesions screened	4	7	0	84	95	2	5	6	23	36
New PKDL cases detected	0	5	0	60	65	0	2	0	7	9
*Index case based approach*										
# old VL index cases reported by PHC	80	58	19	79	236	30	16	5	87	138
New VL cases detected in focal search around index case (rK39 +ve)	4	3	0	5	12	0	1	0	3	4
New PKDL cases detected in focal search	0	0	0	15	15	0	0	0	0	0
*Incentive based approach (over 4 months)*										
New VL cases detected by incentives	15	4	4		23					
Total population screened in incentive approach study area (HH survey)	42874	44090	34015		120979					
Old VL cases (1 yr) detected in HH screening in incentive area	19	6	4		29					
New VL cases^5^ (rK39 +ve) detected in HH screening in incentive area	19	6	4		29					

1 Old case defined as case detected by passive case detection/routine surveillance but not necessarily notified.

2 New case defined as fever more than 14 days + splenomegaly + rK39 test positive.

3 New cases detected by blanket approach includes the new cases detected by the preceding camp approach.

4 Old cases detected by blanket approach includes the old cases detected by the preceding camp approach.

5 New VL cases detected in household survey includes the new VL cases detected by the incentive based approach.

#### Blanket approach

Following the screening camps, all households in the same villages were screened by house to house visits. A total of 165850 individuals were screened for VL/PKDL in the 1^st^ round of the blanket household survey. Overall, out of a total of 549 chronic fever patients, an additional 6 new hitherto undiagnosed patients had spleen enlargement and tested positive with the rK39 test with 1 new VL case in Saran, India and 5 new cases in Bangladesh. No new VL cases were detected in the blanket household survey additional to those already detected in the screening camps in Muzaffarpur, India and Sarlahi, Nepal (table 1). The percentage of chronic fever cases testing positive for VL in the blanket household survey was 10% and 1.9% in India, 6.2% in Nepal and 5.5% in Bangladesh in the 1^st^ round.

#### Index case based approach

Overall, a total of 236 index cases (VL cases reported by PHC in the past 1 year) were identified in the study areas. A simulation exercise of a focal search of households within 50 m radius of the index cases yielded a total of 12 new cases of hitherto undiagnosed VL (table 1).

#### Incentive based approach

The incentive approach was applied only in the Indian and Nepal sites in a separate PHC/Union area. Basic health workers/volunteers identified over a period of 6 months, a total of 23 hitherto undiagnosed new VL cases out of a total of 29 new VL cases, the 6 additional cases being detected in the same communities through a cross sectional house to house screening at the end of the period (table 1).

### Sensitivity of different strategies for active VL case detection

Sensitivity for the camp, index and incentive approach was calculated separately as percentage of new VL cases identified by each of the approaches divided by the new VL cases identified by the ‘gold standard’ blanket approach. Overall, in the 1^st^ round, the camp approach detected 78.6% of all new VL cases in the study villages: 64.3% in Bangladesh, 83.3% in Saran, India but 100% in Muzaffarpur, India and Sarlahi, Nepal (table 2).

**Table 2:** Sensitivity (95% CI) of camp, index and incentive approach with reference to blanket approach for detecting VL and/or PKDL cases.

	Round 1					Round 2				
	Saran India	M'pur India	Sarlahi Nepal	M'sinh B'desh	Overall	Saran India	M'pur India	Sarlahi Nepal	M'sinh B'desh	Overall
*Camp*										
VL(95% CI)	83.3% (5/6)(35.8–99.5)	100% (3/3)(29.2–100)	100% (5/5)(47.8–100)	64.3% (9/14)(35.1–87.2)	78.6% (22/28)(59.0–91.7)	62.5% (5/8)(24.4–91.4)	100% (2/2)(15.8–100)	100% (3/3)(29.2–100)	28.6% (2/7)(3.6–70.9)	60% (12/20)(36.0–80.8)
PKDL(95% CI)	---	0% (0/5)(0–52.2)	---	70.0% (42/60)(56.7–81.1)	64.6% (42/65)(51.7–76.1)	---	0% (0/2)(0–84.1)	---	42.9% (3/7)(9.8–81.5)	33.3% (3/9)(7.4–70.0)
*Index*										
VL(95% CI)	66.7% (4/6)(22.2–95.6)	100% (3/3)(29.2–100)	0% (0/5)(0–52.1)	35.7% (5/14)(12.7–64.8)	42.9% (12/28)(24.4–62.8)	0% (0/8)(0–36.9)	50% (1/2)(1.2–98.7)	0% (0/3)(0–70.7)	42.9% (3/7)(9.8–81.5)	20% (4/20)(5.7–43.6)
PKDL(95% CI)	---	0% (0/5)(0–52.2)	---	25.0% (15/60)(14.7–35.8)	23.1% (15/65)(13.5–35.2)	---	0% (0/2)(0–84.1)	---	0% (0/7)(0–40.9)	0% (0/9)(0–33.6)
*Incentive*										
VL(95% CI)	78.9% (15/19)(54.4–93.9)	66.7% (4/6)(22.2–95.6)	100% (4/4)(39.7–100)	NA	79.3% (23/29)(60.2–92.0)	NA	NA	NA	NA	NA
PKDL(95% CI)	---	---	---		---					

The index case approach detected less than half (42.9%) of new VL cases detected by the blanket approach (35.7% in Bangladesh, 66.7% in Saran, India) which covered a larger area than the 50 meter around index case houses. No new VL cases were detected in households situated within 50 m radius of any of the 19 index cases identified in Nepal (table 2) in contrast to Muzaffarpur in India where the index case approach was able to detect all the 3 new VL cases identified by the blanket approach in the community.

Overall, the incentive approach was able to detect 79.3% of new VL cases in the community (66.7% and 78.9% in the Indian sites and 100% in Nepal; table 2).

### Periodicity of ACD strategies

The 2^nd^ round of screening was implemented 4–6 months after the 1^st^ round. The yield of new cases detected through the camp approach was, with the exception of Saran, India, in all study sites lower in the 2^nd^ round as compared to the 1^st^ round (average number of patients detected with fever more than 14 days ranged from 1.6 and 3.5 patients per camp in India to 7.5 in Nepal and 4.6 patients in Bangladesh). The percent increase of VL cases by adding ACD to PCD was lower in the 2^nd^ round (see below). Overall, out of a total of 196 fever cases detected, only 12 hitherto undiagnosed VL patients were identified which is 6.1% of the chronic fever cases.

Similarly, in the blanket approach of round 2, out of a total of 409 chronic fever cases, 20 hitherto undiagnosed VL cases were detected (4.9%; table 1).

A total of 20 new VL cases were identified in the 2^nd^ screening round (India 8 in Saran and 2 in Muzaffarpur; Nepal – 3; and Bangladesh – 7) compared to 28 new VL cases in the 1^st^ round. Regarding the sensitivity of different ACD approaches, there was a non-significant decrease from the first to the second round (table 2); the sensitivity of the camp approach was 60% and of the index case approach 20%.

### VL disease burden in the study areas

The VL disease burden, estimated as the annual VL incidence by adding the “old” cases, which have been detected by PCD during the preceding 12 months and “new” cases detected by the blanket approach (which includes the case detected by camps) was similar across all sites: 23.4 per 10,000 in Bangladesh; 27.1 and 27.8 in the Indian sites and 28.8 per 10 000 in Nepal (which was due to a VL outbreak in one of the study villages with 96 cases in the preceding year). The percent increase of VL cases (i.e. ACD cases (x100)/PCD cases) was substantial with a 115% overall increase of case numbers (22x100/19) when adding ACD to PCD. In the second round the percent increase was less with 54.5% (12×100/22).

### PKDL detection

In the 1^st^ round, a total of 42 new PKDL cases (all from Bangladesh) were detected by the camp approach and 23 additional new PKDL cases (Bangladesh – 18; Muzaffarpur, India – 5) in the subsequent blanket household screening. Interestingly, the index case approach was able to detect 15 of these new PKDL cases in the focal search around any of the VL index cases. The incentive based approach (not done in Bangladesh) did not yield any new PKDL case. A total of 9 new PKDL cases (Muzaffarpur, India – 2; Bangladesh – 7) were detected in the 2^nd^ round using the blanket approach. The Nepal and the Saran, India site did not detect any new PKDL case during both rounds.

The point prevalence of PKDL (known + new PKDL cases based on the blanket approach) is estimated to be about 1.9 and 18.2 per 10,000 in India and Bangladesh respectively.

The camp approach in Bangladesh was able to detect 70% (42/60) of all new PKDL cases in the community compared to the blanket approach in the 1^st^ round and 42.9% (3/7) in the 2^nd^ round.

### Effort and costs of ACD

In the 1^st^ round, the effort to detect a new VL case through the camp approach was highest in India (0.14 and 0.26 new VL cases detected per camp) compared to 0.60 and 0.83 cases per camp in Bangladesh and Nepal respectively.

In the index case approach the search in the neighborhood of an index case yielded about 0.05 to 0.06 new VL cases per focal search in India and Bangladesh respectively. Estimates for Nepal could not be determined as there were no new cases detected through the index case approach.

The number of households to be screened to detect one new VL case was higher for the blanket approach (1092 households per 1 new VL case; range 654 to 2433 households (non-incentive approach area) and 978 households, range 594 to 1888 households, in the incentive approach areas) than for the geographically restricted index approach areas where 648 households (range 174 to 1000 households) would have to be screened for detecting one new VL case (table 3).

**Table 3:** Efforts required for different ACD strategies.

	Round 1					Round 2				
	SaranIndia	M'purIndia	SarlahiNepal	M'sinhB'desh	Overall	SaranIndia	M'purIndia	SarlahiNepal	M'sinhB'desh	Overall
Mean # of new VL cases detected per camp	0.26	0.14	0.83	0.6	0.36	0.28	0.14	0.50	0.14	0.23
Mean # of new VL cases detected per index case	0.05	0.05	---	0.06	0.05	---	0.06	---	0.03	0.03
# households screened to detect a new VL case										
Index case based approach	1000	967	---	174	648	---	800	---	249	387
Incentive based approach	594	1888	1511	NA	978	NA	NA	NA	NA	NA
Blanket approach	1326	2433	1234	654	1092	991	3672	1991	1305	1519

The average cost (including costs for training, preparation and conduct) for a camp in the 1^st^ round, ranged from USD 85.8 in Muzaffarpur, India to USD 195 in Nepal (table 4). The cost for detecting a new VL case by the camp approach ranged from USD 21.72 in Muzaffarpur, India to USD 661 in Saran, India. The estimated cost for detecting a new VL case by the index case approach (using cost simulations, see methods) ranged from about USD 149.10 in Bangladesh to about USD 200 in the Indian sites. The cost for detecting a new VL case by the incentive based approach ranged from USD 50 and 95 in India to USD 543.25 in Nepal. Similarly, the cost for detecting a new VL case by the blanket approach ranged from USD 112 in Muzaffarpur, India to USD 629 in Nepal.

**Table 4:** Direct costs of different ACD strategies.

	Round 1					Round 2				
	SaranIndia	M'purIndia	SarlahiNepal	M'sinhB'desh	Overall	SaranIndia	M'purIndia	SarlahiNepal	M'sinhB'desh	Overall
*Camp approach*										
Training cost	267	81.33	114	288.32	187.66	267	88.89	96.66	58.69	127.81
Travel, Preparation cost	2026	669.11	184	465.46	836.14	1982	595.56	168	394.74	785.07
Allowance cost for camp	1014	786.67	836	422.34	764.75	961	733.33	886.67	398.25	744.81
Materials cost	0	265.69	36	426.16	181.96	0	89.29	36	35.77	40.26
Total (camp approach)	3307	1802.80	1170	1602.30	1970.53	3210	1507.07	1187.33	887.50	1697.98
*Index case approach*										
Training cost	93		0	14.60	35.86	93	---	---	11.68	52.34
Travel, Preparation cost	333		176	353.77	287.59	333	---	---	311.60	322.30
Allowance cost for screening	374		384	287.30	348.43	374	---	---	287.30	330.65
Materials cost	0		2.67	89.60	30.75	0	---	---	26.32	13.16
Total (index case approach)	800	572^1^	562.65	745.53	702.72	800	309^1^	---2	636.90	718.45
*Incentive based approach*										
Training cost	511	181	793.33		495.11					
Travel, Preparation cost	0	0	113.33		37.77					
Allowance cost for screening	233	164	1032		476.33					
Materials cost	0	35	234.67		89.89					
Total (incentive based approach)	744	380	2173.33	NA	1099.11					
*Blanket approach*										
Training cost	889	38	188	52.41	291.85	889	90.67	180	0	289.91
Travel, Preparation cost	1422	1776	97	1057.10	1088.03	1422	1866.67	95	219.71	900.84
Allowance cost for screening	933	7200	2834	1328.76	3073.94	933	2411.11	2926	646.42	1729.13
Materials cost	0	267	26	749.20	260.55	0	288.89	30	586.20	226.27
Total (blanket approach)	3244	9281	3145	3187.40	4714.35	3244	4657.33	3231	1452.33	3146.17
Cost per new VL case detected (USD)										
Camp approach	661	21.72	234	178.03	273.68	642	22.83	395.78	443.75	376.09
Index case based approach	200	191	---	149.10	180.03	---	---	---	212.30	212.30
Incentive based approach	50	95	543.25	NA	229.41	NA	NA	NA	NA	NA
Blanket approach	541	112	629	227.67	377.41	405	202.49	1077	207.47	472.99
Cost per camp (USD)	174	85.8	195	106.8	129.2	178.3	107.6	197.8	63.4	130.6

1 Detailed costs break up for Index case approach for Muzaffarpur, India not available.

2 Costs for Index case based approach for Nepal has been estimated over the entire study period and not by round.

## Discussion

Although we did not take a representative sample of our study populations, it became clear that the annual VL incidence in endemic districts in India and Bangladesh continues to be very high – more than 20 times the elimination target of 1 per 10,000 to be achieved by 2015 and in the range of previous studies [8], [9]. The burden of VL disease is grossly underestimated [10], [11] by the health systems in the Indian sub-continent due to over-reliability on passive surveillance. Active case detection strategies (household screening and index case based screening) were shown to provide a more realistic representation of the VL burden and are able to improve early diagnosis and potentially treatment of VL [12].This has been reconfirmed in our study where the increase of VL cases by adding ACD to PCD was more than double in the first screening round.

In the present study we could show that the blanket approach of household screening for VL yielded the highest number of new cases, though this ‘gold standard’ for active case detection was expensive and will be difficult to sustain by health systems. Moreover, the effort to conduct the blanket approach is much higher in terms of training manpower for the screening and supervision to ensure the quality of screening and subsequent actions such as spleen examination and Rapid Diagnostic Tests (RDTs).

The camp approach has been extensively used in public health care such as in pulse polio immunization, vitamin A prophylaxis and other public health interventions. This strategy is being tested for the first time in the context of disease surveillance for early diagnosis and treatment of VL. The camp approach, though largely standardized in our study, was implemented with some local variation in India, Nepal and Bangladesh and was successful in detecting three quarter of the unrecognized VL burden in the community. Only approximately 5% of patients with chronic fever had VL and this is a similar proportion of patients with chronic cough that have tuberculosis [13]. This means that it is cost-effective to have trained health staff in the team who can complete the VL diagnosis by doing spleen examination and the RDT. Although we did not compare different delivery mechanisms of chronic fever camps, it can be assumed that camp attendance can be improved by better pre-camp preparation to overcome an indifferent attitude of grass-root level village health workers and negative community attitudes towards public health interventions. The sensitivity of the fever camps for identifying new cases, although of acceptable levels in our study, may also be improved by using better communication skills to increase the demand of camp services. The costs of the camp approach were reasonable in our study. However, areas for savings can possibly be identified. Additional preparatory and planning efforts could also increment program costs.

The index case approach as conducted in our study limited the search for new cases to a 50 m radius around the houses of an index case. This is based on the study by Bern et al [14] who found the proximity to a previous VL case to be a high risk factor for a new infection and on unpublished data by Mondal et al. (personal communication) who found a sharp decrease of VL seroprevalence from Index case houses beyond a radius of 50 m. However, as long as we do not have reliable information on the flight range of vectors and the role of subclinical VL cases in the transmission dynamics, we will not be able to provide clear guidance for where and when to search for secondary cases around an index case. In our study the index case approach was able to identify less than half the VL burden in the community. This was mainly due to the fact that the other newly detected VL cases by house to house screening were living beyond the 50 m radius limit indicating the need for extending the search area in the index case approach of a public health program. A special case was Nepal where in one village, due to an earlier VL outbreak, many index cases were located and extensive case tracing had been done previously, so that no new cases could be detected. Though the pattern is not definitive, it appears that the index case approach performs reasonably well in moderate (Bangladesh) to high (India) VL endemic areas but further research is required for confirmation. The estimated costs of the index case approach were comparatively low though the approach needs to be modified to include geographical areas beyond the 50 m radius around index case houses. It will also be necessary to establish whether the chronic fever patient has to be sent to the health services for further assessment or can receive a full diagnosis, including spleen examination and RDT by a skilled health worker in the home village.

The incentive based approach was able to detect 80% of the VL burden in the community, the highest (100%) being in Nepal where the VL endemicity is relatively low [5]. Though not definitive, this suggests that the incentive based approach works well in low VL endemic areas. Incentives have been tried extensively in public health to improve utilization of services such as contraception, tuberculosis treatment and others. India has recently introduced performance based incentives into their health systems to achieve health care targets. The costs of incentive based approach for VL disease surveillance was lowest (except in Nepal) in our study yet effective in low disease burden settings. Most economic burden studies have focused on costs of VL patient management [15]–[17] and not on case detection. Further research is needed to assess cost effectiveness of the full VL elimination program strategies.

The study also provides insights into the periodicity with which active case detection should be applied. The second ACD rounds for camp and index case approaches showed a much lower percent increase of newly detected VL cases compared to the 1^st^ round and likewise the number of VL cases detected per camp decrease from 0.36 (1^st^ round) to 0.23 (2^nd^ round). Likewise in the index case approach the number of new cases per index case was 1.66 times higher in the 1^st^ round (0.05 new VL cases per index case) than in the 2^nd^ round (0.03 new VL cases per index case). On the other hand, the number of newly detected cases in the second screening round by the camp approach was still substantial, so that affordability and staff availability will be important for the decision if ACD through camps should be done during the current VL elimination campaign once or twice per year in the same villages. Further research on different ACD delivery mechanisms and their costs can provide additional support for rational decision making.

The study provided further evidence on the huge burden of PKDL disease in the community especially in Bangladesh [18] and the ability of ACD to shed light on this largely unrecognized health problem. Further research is needed to better understand the low PKDL prevalence in India and Nepal and to determine cost effective treatment interventions.

In conclusion, the study provides evidence on the effectiveness and costs of different active case detection strategies. Preliminary evidence suggests use of the camp approach in high VL endemic settings, index case surveillance in high to moderate VL endemic settings and incentive based surveillance in low VL endemic settings. The study findings are useful for Country VL Elimination Programs to choose the most appropriate ACD method or tool mix for their communities, depending on the level of VL endemicity, feasibility of implementing these strategies and affordability within the context of their health systems.

## Supporting Information

STROBE checklist (0.09 MB DOC)
